# The Association between ESR and CRP and Systemic Hypertension in Sarcoidosis

**DOI:** 10.1155/2016/2402515

**Published:** 2016-06-28

**Authors:** Mehdi Mirsaeidi, Hesham R. Omar, Golnaz Ebrahimi, Micheal Campos

**Affiliations:** ^1^Division of Pulmonary, Critical Care, Sleep and Allergy, Department of Medicine, University of Miami, FL, USA; ^2^Internal Medicine Department, Mercy Medical Center, Clinton, IA, USA

## Abstract

*Introduction.* The association between the level of systemic inflammation and systemic hypertension (sHTN) among subjects with sarcoidosis has not been previously explored.* Methods.* A retrospective study was conducted to investigate the relation between the level of systemic inflammation in sarcoidosis, measured by various serum inflammatory markers, and sHTN.* Results.* Among a total of 108 cases with sarcoidosis (mean age: 53.4 years, 76.9% females), 55 (50.9%) had sHTN and 53 (49.1%) were normotensive. ESR was highly associated with sHTN. The patients with sHTN had higher mean ESR levels compared with normotensives (48.8 ± 35 versus 23.2 ± 27 mm/hr, resp.; *P* = 0.001). ROC curve analysis for ESR revealed an AUC value of 0.795 (95% CI: 0.692–0.897; *P* = 0.0001). With regard to CRP, there was a trend towards higher mean values in sHTN group (3.4 versus 1.7 mg/L; *P* = 0.067) and significantly higher prevalence of sHTN in the highest CRP quartile compared to the lowest one (69.6% versus 30%; OR 4.95; *P* = 0.017). ROC curve analysis for CRP revealed an AUC value of 0.644 (95% CI: 0.518–0.769; *P* = 0.03). On multivariate analysis, ESR and the CRP remained independent predictors for sHTN among subjects with sarcoidosis.* Conclusion.* Systemic inflammation is associated with the presence of sHTN in sarcoidosis.

## 1. Introduction

Inflammation is recognized as a main pathogenic element in the development of pulmonary arterial hypertension (PH) [1, 2] with several studies showing a significant association between systemic inflammatory markers and PH [3, 4]. Inflammation is also a precursor for the development and progression of atherosclerosis and systemic hypertension (sHTN). The association between C-reactive protein (CRP) and sHTN has been established in multiple cross-sectional studies [5–9], in particular after the advent of high sensitivity CRP (hs-CRP) assays capable of detecting levels that were earlier considered to be normal. Higher levels of CRP may contribute to the development of sHTN through the reduction of endothelium-dependant relaxation by reducing nitric oxide production in endothelial cells [10, 11], resulting in vasoconstriction and increased production of endothelin 1 [12]. CRP may also promote atherosclerosis through upregulating angiotensin type 1 receptor expression [13]. Another marker of systemic inflammation is albumin which is an acute phase reactant that is influenced by multiple factors mainly including malnutrition and inflammation which may reduce albumin levels irrespective of the patient's nutritional status [14, 15]. Other inflammatory markers include erythrocyte sedimentation rate (ESR), ferritin, angiotensin-converting enzyme (ACE), and 25 OH vitamin D.

Sarcoidosis is a systemic inflammatory disease that is associated with higher incidence of PH not only in cases with evidence of advanced parenchymal fibrosis, but also in cases without significant interstitial lung disease. In fact, anti-inflammatory therapies can have an impact on the progression of sarcoidosis-associated PH [16]. The relationship between chronic granulomatous inflammatory disease and systemic hypertension had not been previously explored. The aim of this study was to investigate the relation between the degree of systemic inflammation measured by various inflammatory markers and sHTN in sarcoidosis patients.

## 2. Materials and Methods

This is a retrospective observational study of consecutive adult subjects >18 years who were diagnosed with sarcoidosis at the University of Illinois at Chicago between January 2010 and January 2015. The diagnosis of sarcoidosis was made according to the European Respiratory Society (ERS), American Thoracic Society (ATS), and World Association of Sarcoidosis and other Granulomatous Disorders (WASOG) criteria [17]. The Institutional Review Board of the University of Illinois at Chicago approved the study and waived the need for patient consent (approval number of 20130195001). Cases were divided into two groups based on the presence or absence of an underlying diagnosis of sHTN. The diagnosis of sHTN was made according to the seventh report of the joint national committee guidelines (JNC 7) if the blood pressure was ≥140/90 mmHg [18] in at least two separate visits or if the patient has been taking antihypertensive medication(s).

The inflammatory markers evaluated in this study were serum ESR, CRP, albumin, ferritin, ACE, and 25 OH vitamin D. Patients had been routinely tested for these inflammatory biomarkers to track the treatment response in several visits during a year. We used the mean recorded values for ESR, CRP albumin, ferritin, and 25 OH vitamin D. Data on patient demographic characteristics, medical comorbidities, clinical and laboratory variables, pulmonary function test (PFT), treatment, and outcome were collected. Blood sampling for biomarkers was performed by venipuncture. ESR was read after 30 minutes of bed rest and the reference range for it is 0–20 mm/hr. The blood levels of CRP were measured by immunonephelometry and the reference range for CRP was 0.5–2 mg/L. Albumin is measured by protein electrophoresis and reported in gm/dL. Serum 25 (OH) vitamin D was assayed using a competitive protein binding assay employing automated chemiluminescence and expressed in ng/mL. Serum ferritin concentration is determined by enzyme immunoassay and expressed in *μ*g/L.

### 2.1. Statistical Analysis

Primary analysis was to compare biomarker values between sarcoidosis cases with and without sHTN. Continuous variables are expressed as mean ± standard deviation or median and interquartile range (IQR) and compared using Student *t*-test. Categorical variables are described as counts and percentages and compared using the Chi-square test. In order to study the effect of increase ESR, CRP, and albumin level on the prevalence of sHTN, we have divided the cohort into 4 groups according to the 4 quartiles of the ESR, CRP, and albumin, and the Cochran-Mantel-Haenszel common odds ratio test was used to evaluate the odds ratio (OR) and *P* value for the prevalence of hypertension in the 2nd, 3rd, and 4th ESR and CRP quartiles using the first quartile as the comparator, or the 1st, 2nd, and 3rd quartile using the fourth quartile as a comparator in the case of albumin. Chi-square test was utilized to detect whether the trend in sHTN prevalence across the four ESR and CRP quartiles was significant or not. A ROC (receiver operating characteristics) curve analysis was implemented to detect the ideal cutoff value of ESR, CRP, and albumin that yields the highest sensitivity and specificity for predicting sHTN and to calculate the area under the curve (AUC). In order to examine independent predictors for sHTN in sarcoidosis patients and avoid confounding factors, we used a multivariate logistic regression model with backward stepwise elimination. The variables used in the model were either those that achieved statistical significance at *P* < 0.01 upon univariate analysis or variables considered to be clinically relevant. A *P* value less than 0.05 is considered statistically significant. All statistical analyses were 2-sided. Data were analyzed using IBM SPSS 21.0 statistical software (IBM SPSS Version 21.0, Armonk, NY).

## 3. Results

### 3.1. Patient Characteristics

A total of 108 cases diagnosed with sarcoidosis were included in the study, among which 55 patients (50.9%) had sHTN and 53 (49.1%) were normotensive. The mean age of the study population was 53.4 ± 9.4 years, 76.9% were females, 70% were African Americans, and the average duration of sarcoidosis was 12 years. The results of univariate comparison of baseline demographics, clinical and laboratory characteristics, PFT, echocardiographic data, and treatment in sarcoidosis subjects with and without sHTN are summarized in Table 1. Compared with normotensive patients, those with sHTN were older (56 versus 50.6 years, *P* = 0.002), had a higher body mass index (33.5 versus 30.2 Kg/m^2^, *P* = 0.037), had a higher proportion of African American race (89.1% versus 50.9%, *P* = 0.0001), and had a longer mean duration of sarcoidosis (14.3 versus 10 days, *P* = 0.014). Pulmonary-wise, subjects with sHTN have a higher proportion of PH (37% versus 15.1%, *P* = 0.012), were more likely to complain of dyspnea (62.7% versus 43.1%, *P* = 0.049), and had significantly lower lung function values (FVC%, FEV1%, TLC%, and DLCO). Most of the study cohort (83%) was receiving oral steroids, 43.9% were on disease modifying antirheumatic drugs, 29.6% were on methotrexate, and 4.7% were on azathioprine. At 5 years, 84 cases were still available for follow-up information while the rest were lost to follow-up.

### 3.2. Association between ESR and Systemic Hypertension

**Figure 1:**
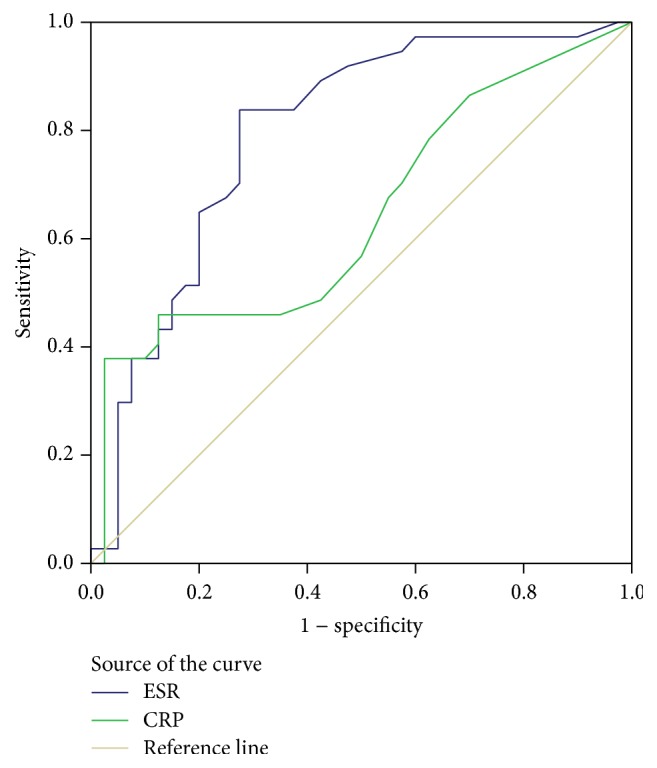
Receiver operating characteristic (ROC) curve to detect the best cutoff value for ESR and CRP in the prediction of systemic hypertension in sarcoidosis patients. ESR had an AUC value of 0.795 (95% CI 0.692–0.897, *P* = 0.0001). A cutoff value for ESR of 30 mm/hr yielded a 62% sensitivity and 80% specificity for predicting systemic hypertension in sarcoidosis patients. With regard to CRP, the AUC value is 0.644 (95% CI 0.518–0.769, *P* = 0.03). A cutoff value for CRP of 3 mg/L yielded a 37% sensitivity and 95% specificity for predicting systemic hypertension in sarcoidosis patients.

Subjects with sHTN had significantly higher mean and median ESR levels (Table 2). Furthermore, the prevalence of sHTN significantly increased from the 1st to the 4th ESR quartile, with crude prevalence rates of sHTN being 10%, 40.9%, 63.2%, and 75%, respectively (*P* value for the trend = 0.0001). A total of 23 subjects (67.6%) in the sHTN group had ESR levels in the 3rd or 4th higher quartiles, compared with only 11 (32.4%) of the subjects without sHTN (*P* = 0.002). Using the Cochran-Mantel-Haenszel method, compared to the lowest ESR quartile, the OR to have sHTN were 6.2 (*P* = 0.034), 15.4 (*P* = 0.002), and 33.8 (*P* = 0.0001) for the 2nd, 3rd, and 4th ESR quartiles, respectively. ROC curve analysis performed to detect the best cutoff value for ESR in predicting sHTN in sarcoidosis patients revealed an AUC value of 0.795 (95% CI 0.692–0.897, *P* = 0.0001) (Figure 1). A cutoff value for ESR of 30 mm/hr yielded a 62% sensitivity and 80% specificity for predicting the presence of sHTN in this sarcoidosis patient cohort.

### 3.3. Association between CRP and Systemic Hypertension

Subjects with sHTN had a trend towards higher median and interquartile range of CRP levels [1.1 (0.65, 3.65) versus 0.9 (0.5, 1.8), resp., *P* = 0.067] compared with normotensive subjects as shown in Table 2. On dichotomizing CRP values, sarcoidosis patients with sHTN had a significantly higher frequency of CRP >2 mg/L (*P* = 0.003), CRP >2.5 mg/L (*P* = 0.004), CRP >3 mg/L (*P* = 0.001), and CRP >3.5 mg/L (*P* = 0.01). When we dichotomized the cohort according to CRP levels, the prevalence of sHTN was 88.9% when CRP >3 mg/L versus 45% when CRP <1 mg/dL (univariate OR for the prevalence of sHTN when CRP >3 mg/dL versus CRP <1 mg/dL 9.778, 95% CI 1.981–48.261, *P* = 0.005). The crude prevalence of sHTN increased from 30% in the first CRP quartile to 76.2% in the 4th quartile (OR 6.933, *P* = 0.006). *P* value for the trend in sHTN prevalence across the 4 CRP quartiles was 0.003. ROC curve analysis for CRP revealed an AUC value of 0.644 (95% CI 0.518–0.769, *P* = 0.03) (Figure 1) and a cutoff value of 3 mg/L yielded a 37% sensitivity and 95% specificity for predicting the presence of sHTN in this cohort of sarcoidosis subjects.

### 3.4. Association between Hypoalbuminemia and Systemic Hypertension

**Figure 2:**
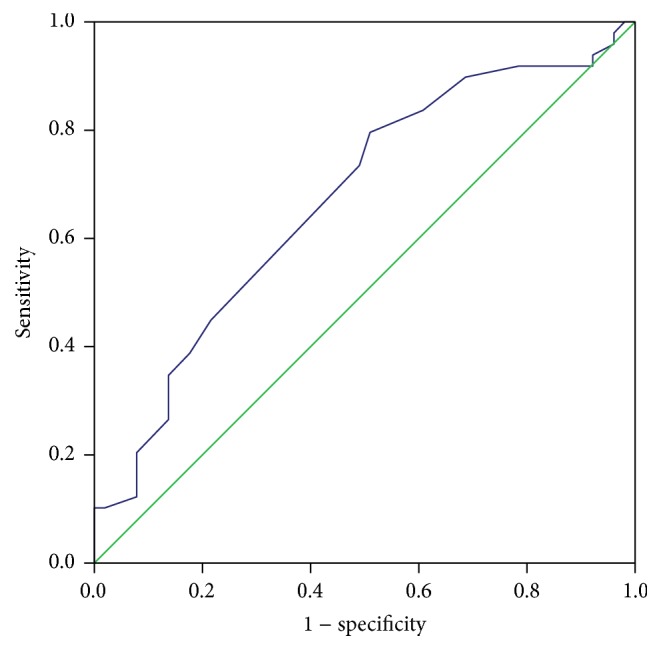
Receiver operating characteristic (ROC) curve to detect the best cutoff value for albumin in the prediction of systemic hypertension in sarcoidosis patients. AUC value of 0.670 (95% CI 0.564–0.776, *P* = 0.003). An albumin level < 3.3 gm/dL yielded a 35% sensitivity and 87% specificity for predicting systemic hypertension in sarcoidosis patients.

The median and interquartile range (IQR) of albumin for the entire cohort was 3.7 g/dL (IQR 3.4−4 gm/dL). Sarcoidosis patients with sHTN had a significantly lower mean albumin level compared with normotensives (3.44 versus 3.77 gm/dL, resp., *P* = 0.004). Opposite to what was observed with ESR and CRP, the prevalence of hypertension significantly decreased along albumin quartiles (1st quartile < 3.4, 2nd quartile 3.4–3.69, 3rd quartile 3.7–4, and 4th quartile > 4 gm/dL), with the crude prevalence rates of sHTN being 70.8%, 52.6%, 48.3%, and 28.6% for the 1st, 2nd, 3rd, and 4th quartile, respectively (*P* value for the trend = 0.025). Using the Cochran-Mantel-Haenszel method, the OR to have sHTN in the first albumin quartile compared with the 4th albumin quartile was 5.77 (95% CI 1.725–19.287, *P* = 0.004). ROC curve analysis revealed an AUC value of 0.670 (95% CI 0.564–0.776, *P* = 0.003) (Figure 2). An albumin level < 3.3 gm/dL yielded a 35% sensitivity and 87% specificity for predicting systemic hypertension in this sarcoidosis cohort.

### 3.5. Relationship between Other Inflammatory Markers and Systemic Hypertension

We have also evaluated the relationship between sHTN and other nontraditional markers of inflammation including ferritin, ACE, and 25-hydroxy vitamin D. On univariate analysis, there was no significant difference between those with sHTN and normotensive subjects with regard to the mean ferritin level (245 versus 83 *μ*g/L, *P* = 0.262), mean ACE level (52.9 versus 71.4 U/L, *P* = 0.121), and mean 25 OH vitamin D (15.7 versus 16.5 ng/mL, *P* = 0.657).

### 3.6. Multivariable Logistic Regression Analysis

**Figure 3:**
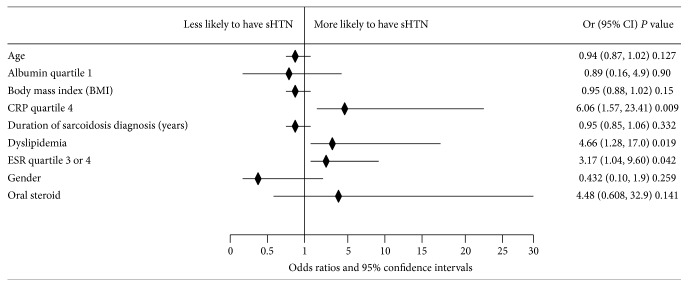
Forest plot of multivariate logistic regression analysis demonstrating independent factors associated with systemic hypertension in sarcoidosis patients. Hosmer and Lemeshow *P* = 0.775.

Multivariate logistic regression analysis with backward stepwise elimination did not change the positive dose-response relationship between the ESR and CRP levels and the prevalence of sHTN. In the multivariate logistic regression model (*N* = 70, Nagelkerke *R* ^2^ = 0.332, and *P* from Hosmer and Lemeshow = 0.775), we found that having ESR levels in the 3rd and 4th quartiles combined (OR 3.165, 95% confidence interval 1.044–9.593, *P* = 0.042) and CRP levels in the 4th quartile (OR 6.057, 95% confidence interval 1.567–23.415, *P* = 0.009) remained independent predictors for a subject with sarcoidosis to have sHTN after adjustment for age, sex, body mass index, dyslipidemia, sarcoidosis duration, the use of oral steroids, and hypoalbuminemia. We have not found a relationship between other inflammatory markers such as ferritin, 25 OH vitamin D level, and neutrophil-to-lymphocyte ratio and sHTN on univariate or multivariable analysis. The variables used in the multivariate model are shown as a Forest plot (Figure 3).

## 4. Discussion

We were able to show in this retrospective analysis that an independent relationship exists between the degree of systemic inflammation and sHTN in sarcoidosis patients. Previous work showed that ESR [19, 20] and CRP [20] levels are higher in sarcoidosis patients compared to controls. We observed that, among sarcoidosis subjects, ESR and CRP levels are almost twice as high in hypertensive subjects compared with normotensive patients (48.8 versus 23.2 mm/hr, *P* = 0.001 for ESR, and 3.4 versus 1.7, *P* = 0.067 for CRP). Furthermore, the link between sHTN and inflammation is supported by the stepwise increase in sHTN prevalence across quartiles of both ESR and CRP. These results imply that a more intense inflammatory response may have an important pathophysiologic contribution for the development of sHTN in sarcoidosis. Patients with pulmonary sarcoidosis and systemic hypertension also had significantly higher frequency of pulmonary hypertension (*P* = 0.012) where inflammation is a recognized factor in its pathogenesis.

The association between CRP and sHTN has been previously demonstrated in other studies not related to sarcoidosis. For example, in a cross-sectional study of 300 subjects ≥30 years old, Bautista and colleagues found that the prevalence of sHTN was 1.56 times (95% CI 1.14–2.13, *P* = 0.005) higher in subjects in the fourth quartile of CRP as compared to subjects in the first quartile [21]. In a later cross-sectional survey of 8347 apparently healthy Koreans, Sung and colleagues found a significant positive association between the blood pressure and CRP level (*P* < 0.0001) and after adjustment for confounding variables, the prevalence of hypertension by CRP was significantly higher in subjects in the second, third, and fourth quartiles of CRP compared to subjects in the first quartile [22]. Furthermore CRP was found to be a predictor of future development of hypertension. In an analysis of the Women's Health Study [23], 20,525 females with initially normal blood pressure were followed up for 7.8 years and CRP was found to be significantly associated with an increased risk of developing sHTN over time [24]. Furthermore, Magen and colleagues found a significant positive correlation between systolic blood pressure and CRP levels, with significantly higher levels in the resistant hypertension group compared with the controlled hypertension group (6.9 ± 5.8 versus 4.2 ± 4.8, *P* = 0.021) [25]. These studies utilized the hypothesis that inflammation is important in the pathogenesis of sHTN. In our study, we are trying to show whether systemic inflammation caused by other diseases such as sarcoidosis would predispose to the development of systemic hypertension just like it predisposes these patients to pulmonary hypertension irrespective of lung involvement.

In the above mentioned studies, the association of sHTN with elevated ESR was either insignificant [25], not studied, or not reported. However, we found that ESR gives the highest AUC value in predicting sHTN compared with any of the inflammatory markers studies. Hypoalbuminemia has been studied in multiple respiratory and cardiac diseases and was found to be poor prognostic indicator and an independent predictor of mortality [26]. A large prospective study on emergency department patients showed that the short-term mortality of patients with hypoalbuminemia was three times higher compared with patients with normal albumin after adjusting for several confounders [15]. Albumin is a negative acute phase reactant that is influenced by mainly two factors: patient's nutritional status and inflammation; however, prior studies showed that inflammation reduces albumin levels irrespective of the patient's nutritional state [27]. Serum albumin level is reduced in acute and chronic inflammatory states due to increased degradation from the high catabolic rate in addition to its transudation to extravascular space from increased capillary permeability [15].

The current study is subject to all limitations inherent to nonrandomized observational studies. Our experience is from a single center, the design is retrospective, and the cohort is relatively small due to rarity of the disease. The main study limitation is that we have not taken into account whether the diagnosis of sHTN was made before or after the diagnosis of sarcoidosis and therefore a proportion of these hypertensive patients may have had sHTN prior to and independent of sarcoidosis. However, this is very difficult to control for as both diseases (hypertension and sarcoidosis) are chronic diseases and to know the exact onset of either pathology is not possible. There may have been confounding variables that were not accounted in our analysis. For example, it is known that sarcoidosis patients with active disease have very high levels of ESR and CRP [28]. These inflammatory markers are also significantly elevated in sarcoidosis-associated arthritis [29], erythema nodosum [30], concomitant connective tissue disease, or simultaneous acute infections and these factors have not been accounted for. We have only used sHTN as a diagnosis without staging its severity and therefore we were unable to study the correlation between the level of these inflammatory markers and the severity of sHTN. The OR for the prevalence of sHTN across the quartiles is notably elevated because it is known that, in diseases with high prevalence, the OR may overestimate the effect of exposure (the prevalence of sHTN in this cohort is 50.9%).

We conclude that the level of systemic inflammation in sarcoidosis patients—reflected by a higher ESR, CRP—may be associated with the presence of sHTN. This study provides insight of the role of systemic inflammation in the development of sHTN as an additional complication of the disease, suggesting the importance of a closer follow-up of blood pressure in normotensive sarcoidosis patients with elevated inflammatory markers. The study may also stimulate the conduction of further trials to assess the role of anti-inflammatory drugs in the control and/or regression of sHTN in sarcoidosis subjects who had developed sHTN after the diagnosis of sarcoidosis.

## Figures and Tables

**Table 1:** Demographic and clinical characteristics of the sarcoidosis cohort.

	All subjects (*n* = 108)	Sarcoidosis with HTN (*n* = 55)	Sarcoidosis without HTN (*n* = 53)	*P* value
Baseline demographics and comorbidities				
Age (years, mean ± SD)	53.4 ± 9.4	56 ± 9.2	50.6 ± 8.9	0.002
Female sex% (*n*)	76.9% (83)	76.4% (42)	77.4% (41)	0.902
BMI (Kg/m^2^, mean ± SD)	31.9 ± 8	33.5 ± 8.2	30.2 ± 7.4	0.037
Duration of sarcoidosis (y, mean ± SD)	12.2 ± 9.1	14.3 ± 9.8	10 ± 7.9	0.014
African American% (*n*)	70.4% (76)	89.1% (49)	50.9% (27)	0.0001
Diabetes% (*n*)	31.1% (33)	38.2% (21)	23.5% (12)	0.106
Dyslipidemia% (*n*)	24.3% (26)	33.3% (18)	15.1% (8)	0.031
CKD% (*n*)	3.7% (4)	5.5% (3)	1.9% (1)	0.348
PCI or CABG% (*n*)	1.9% (2)	1.8% (1)	1.9% (1)	0.979
Atrial fibrillation% (*n*)	5.6% (6)	7.3% (4)	3.8% (2)	0.435
CHF% (*n*)	6.6% (7)	9.3% (5)	3.8% (2)	0.276
Pulmonary hypertension% (*n*)	26.2% (28)	37% (20)	15.1% (8)	0.012
Rheumatoid arthritis% (*n*)	3.7% (4)	5.5% (3)	1.9% (1)	0.348
Asthma% (*n*)	26.2% (28)	27.8% (15)	24.5% (13)	0.702
OSA% (*n*)	24.3% (28)	30.9% (17)	17.3% (9)	0.105
Dyspnea% (*n*)	52.9% (54)	62.7% (32)	43.1% (22)	0.049

Pulmonary function tests and echocardiography				
FVC% (mean ± SD)	93.2 ± 20.9	87.7 ± 19.9	99.4 ± 20.4	0.008
FEV1% (mean ± SD)	88 ± 24.9	80.5 ± 23.9	96.7 ± 23.4	0.002
TLC% (mean ± SD)	89.1 ± 15.6	84.7 ± 15	94.1 ± 15.1	0.005
RV% (mean ± SD)	99.4 ± 26.7	98.4 ± 29.4	100.9 ± 22.7	0.711
DLCO% (mean ± SD)	67 ± 20.3	58.8 ± 18.4	76.4 ± 18.4	0.000
EF (mean ± SD)	57.8 ± 4.8	57.1 ± 4.8	58.6 ± 4.7	0.108

Treatment				
Oral steroid% (*n*)	83.5% (86)	88% (44)	79.2% (42)	0.237
DMARD% (*n*)	43.9% (47)	43.6% (24)	44.2% (23)	0.951
Methotrexate% (*n*)	29.6% (32)	25.5% (14)	34% (18)	0.334
Azathioprine% (*n*)	4.7% (5)	3.6% (2)	5.6% (3)	0.620
Lasix% (*n*)	17.6% (19)	23.6% (13)	11.3% (6)	0.099
Warfarin% (*n*)	2.8% (3)	5.5% (3)	0% (0)	0.129
Statin% (*n*)	28.7% (31)	38.2% (21)	18.9% (10)	0.029
ACE or ARB% (*n*)	46.7% (50)	74.1% (40)	18.9% (10)	0.0001
Aspirin	26.9% (29)	34.5% (19)	18.9% (10)	0.069

PHTN: pulmonary hypertension, BMI: body mass index, CKD: chronic kidney disease, PCI: percutaneous coronary intervention, CABG: coronary artery bypass graft, OSA: obstructive sleep apnea, PASP: pulmonary artery systolic pressure, EF: ejection fraction, ESR: erythrocyte sedimentation rate, CRP: C-reactive protein, DMARD: disease modifying antirheumatic drug, ACE: angiotensin converting enzyme, y: year, m: mean, and SD: standard deviation.

**Table 2:** Associations between various inflammatory markers with prevalence of systemic hypertension in sarcoidosis patients.

Inflammatory markers				
	All subjects (*n* = 108)	Sarcoidosis with HTN (*n* = 55)	Sarcoidosis without HTN (*n* = 53)	*P* value
Erythrocyte sedimentation rate				
ESR (mm/hr, mean ± SD)	35.2 ± 33.4	48.8 ± 35	23.2 ± 27	0.001
ESR (mm/hr, median, IQR)	25 (12.5–44)	34.5 (23.8–81)	13 (10–28)	—
ESR quartile 1 (ESR < 12.5 mm/hr), % (*n*)	24.7% (20)	10% (2)	90% (18)	0.001
ESR quartile 2 (ESR 12.5–24 mm/hr), % (*n*)	27.2% (22)	40.9% (9)	59.1% (13)	0.509
ESR quartile 3 (ESR 25–44 mm/hr), % (*n*)	23.5% (19)	63.2% (12)	36.8% (7)	0.11
ESR quartile 4 (ESR > 44 mm/hr), % (*n*)	24.7% (20)	75% (15)	25% (5)	0.006

C-reactive protein				
CRP (mg/L, mean ± SD)	2.5 ± 4.2	3.4 ± 4.5	1.7 ± 3.7	0.067
CRP (mg/L, median, IQR)	1 (0.6, 2.37)	1.1 (0.65, 3.65)	0.9 (0.5, 1.8)	—
CRP > 2% (*n*)	28.9% (24)	45% (18)	14% (6)	0.003
CRP > 2.5% (*n*)	22.9% (19)	37.5% (15)	9.3% (4)	0.004
CRP > 3% (*n*)	20.7% (17)	38.5% (15)	4.7% (2)	0.001
CRP > 3.5% (*n*)	14.5% (12)	27.5% (11)	2.3% (1)	0.01
CRP quartile 1 (CRP < 0.6 mg/dL), % (*n*)	23.8% (20)	30% (6)	70% (14)	0.059
CRP quartile 2 (CRP 0.6–0.99 mg/dL), % (*n*)	28.6% (24)	58.3% (14)	41.7% (10)	0.272
CRP quartile 3 (CRP 1.0–2.37 mg/dL), % (*n*)	22.6% (19)	26.3% (5)	73.7% (14)	0.031
CRP quartile 4 (CRP > 2.37 mg/dL), % (*n*)	25% (21)	76.2% (16)	23.8% (5)	0.006

Albumin				
Albumin (gm/dL, mean ± SD)	3.6 ± 0.58	3.44 ± 0.63	3.77 ± 0.49	0.004
Albumin (gm/dL, median, IQR)	3.7 (3.4–4)	3.6 (3.2–3.8)	3.8 (3.6–4.1)	—
Albumin quartile 1 (albumin < 3.4 gm/dL), % (*n*)	24% (24)	70.8% (17)	29.2% (7)	0.017
Albumin quartile 2 (albumin 3.4–3.69 gm/dL), % (*n*)	19% (19)	52.6% (10)	47.4% (9)	0.725
Albumin quartile 3 (albumin 3.7–4.1 gm/dL), % (*n*)	29% (29)	48.3% (14)	51.7% (15)	0.926
Albumin quartile 4 (albumin > 4.1 gm/dL), % (*n*)	28% (28)	28.6% (8)	71.4% (20)	0.013

Other inflammatory markers				
Ferritin (ng/mL, mean ± SD)	161.4 ± 602	245 ± 857	83 ± 91	0.262
ACE level (U/L, mean ± SD)	63.2 ± 49.5	52.9 ± 55	71.4 ± 43.8	0.121
25 OH vitamin D (*µ*g/L, mean ± SD)	16.1 ± 8.5	15.7 ± 8.7	16.5 ± 8.3	0.657

HTN: systemic hypertension, ESR: erythrocyte sedimentation rate, CRP: C-reactive protein, ACE: angiotensin converting enzyme, m: mean, SD: standard deviation, and IQR: interquartile range.
